# Does increasing overall health expenditure reduce inequality in under 5 mortality rates between provinces in Indonesia?

**DOI:** 10.1093/heapol/czag081

**Published:** 2026-06-18

**Authors:** Iván Ochoa-Moreno, Noémi Kreif, Taufik Hidayat, Sari Kurnia, Budi Hidayat, Susan Griffin

**Affiliations:** Centre for Health Economics, University of York, Heslington, York, YO10 5DD, United Kingdom; Department of Pharmacy, University of Washington School of Pharmacy Health Sciences Building, Room H-362195, 6 NE Pacific Street, Seattle, WA 98195, United States; Center for Health Economics and Policy Studies, Faculty of Public Health, Universitas Indonesia, Building G, 3rd Floor, Room 311, Campus UI, Depok 16424, West Java, Indonesia; Department of Economics, University of Sussex Business School Jubilee Building, Falmer Brighton, BN1 9SL, United Kingdom; Center for Health Economics and Policy Studies, Faculty of Public Health, Universitas Indonesia, Building G, 3rd Floor, Room 311, Campus UI, Depok 16424, West Java, Indonesia; Center for Health Economics and Policy Studies, Faculty of Public Health, Universitas Indonesia, Building G, 3rd Floor, Room 311, Campus UI, Depok 16424, West Java, Indonesia; Centre for Health Economics, University of York, Heslington, York, YO10 5DD, United Kingdom

**Keywords:** health care financing, inequality, mortality, quantile regression, instrumental variables, concentration index, Indonesia

## Abstract

While public health expenditure is widely assumed to reduce health inequalities, empirical evidence from low- and middle-income countries remains limited. This study examines whether increases in public health spending in Indonesia between 2010 and 2017 reduced inequalities in under-five mortality (U5MR). We combined two approaches to assess distributional effects of health spending. First, we used a dynamic panel System-GMM model to estimate the impact of public health expenditure on within-province inequality in U5MR, measured by the concentration index across household wealth quintiles. Second, we applied instrumental variable quantile regression to identify heterogeneous treatment effects across provinces with different baseline mortality burdens. In addition, we estimated the average effect of public health expenditure on U5MR levels and found no statistically significant impact. The results show that higher public health spending increased inequality in U5MR across socioeconomic groups, with wealthier households capturing a disproportionate share of the mortality reductions. By contrast, we find no statistically significant heterogeneity in the impact of spending between high- and low-mortality provinces. These results suggest that increases in spending were not well-targeted to disadvantaged groups or high-mortality regions. Our findings demonstrate that raising overall levels of public spending is not sufficient to reduce health inequalities unless resources are explicitly directed towards disadvantaged groups and underserved areas. This study provides a proof of concept for applying distribution-sensitive methods to assess the equity impact of health financing, offering lessons that remain highly relevant for Indonesia’s post-JKN reforms and contributing to global debates on equity-weighted evaluation, benefit incidence, and the distributional consequences of health spending in LMICs.

Key messages

- This study explores whether increased public health expenditure in Indonesia (2010–17) contributed to reducing inequalities in childhood health—an understudied dimension of Universal Health Coverage.
- Findings show that higher public health expenditure was associated with worsening inequality in child mortality between poorer and wealthier households, suggesting that gains from expenditure disproportionately benefited better-off groups.
- Quantile regression analysis found no significant difference in the effect of spending across provinces with varying mortality burdens, indicating limited responsiveness of resource allocation to regional health needs.
- Simply increasing public health budgets is not enough. Targeted spending strategies are critical to ensure that investments in health reduce disparities and reach the most vulnerable populations.

## Introduction

Universal Healthcare Coverage (UHC) aims not only to improve overall population health but also ensuring that gains in health outcomes are equitably distributed across socioeconomic groups (World Health Organization 2010, Wagstaff et al. 2016). A core principle of UHC is to prioritize health for groups that are more disadvantaged in terms of socioeconomic and health status. Analysing how changes in public health expenditure differentially affect health across socioeconomic groups and between healthier and less healthier regions can provide insights on the impact of health spending on inequality.

Decisions about overall health spending levels are often disconnected from decisions about where these funds are allocated. Understanding how changes in the overall level of funding can affect health inequalities may enable decision-makers to factor such considerations into system level decisions that might more effectively target funding towards specific groups based on how current spending patterns impact on healthcare access and utilization.

Prior research has primarily focused on estimating the average marginal productivity of the health system—the average effect on health of an additional unit of healthcare expenditure—and have found that increased public health spending can reduce absolute levels of mortality and improve access to health services (Gupta et al. 2003, Bokhari et al. 2007, Edney et al. 2022). However, fewer studies explicitly address how such spending affects health inequalities. Wagstaff (2002) and O'Donnell et al. (2008) emphasize the importance of measuring health inequality using tools such as the concentration index (CI), which captures the socioeconomic gradient in health outcomes. Moreover, studies using dynamic panel and quantile regression methods (Grimm 2011, Kotsadam and Østby 2019) show that the average effect of public spending can mask important heterogeneity across population subgroups and regions.

This study seeks to contribute to that literature by exploring methodological approaches to analyse the inequality impacts of public health expenditure. Expanding upon average effects, we aim to estimate impacts across distributions of household wealth and area-level population health. Specifically, in this study we examine differential effects of public health expenditure on health outcomes between the healthiest (e.g. lowest ranked in under-five mortality) and least healthy (highest ranked in under-five mortality) provinces. Furthermore, we analyse how changes in public health expenditure affect relative inequality in under-five mortality rates (U5MR) between poorer and richer households within provinces.

Public health expenditure can influence under-five mortality through various channels. These include direct service provision via government-funded facilities (e.g. staffing, medicines, and utilities), population-level programmes (e.g. immunization campaigns and nutritional support), and financial protection schemes that reduce barriers to accessing care (e.g. health insurance subsidies such as *Askeskin* or *Jamkesda*). The impact on inequality depends on the extent to which these services are accessible and used by lower-income populations. For instance, if additional funds primarily support hospitals in wealthier urban areas or curative services accessed disproportionately by higher-income groups, this may increase existing disparities. Conversely, targeted spending on preventive programmes or rural health infrastructure could reduce inequalities. Our study seeks to estimate who appears to benefit more from this increased public investment—effectively a form of benefit incidence analysis using mortality outcomes.

Indonesia has been reforming its health system over the past decades with overall health status indicators improving significantly at the same time. However, despite improvements Indonesia faced significant health challenges. The country struggled with high rates of communicable diseases, maternal mortality (359 deaths per 100 000 live births), and childhood malnutrition (31% of children under 5 stunted) in 2012. Health indicators showed substantial inequalities across provinces and socioeconomic groups. For instance, in 2012, complete vaccination coverage was 75% for the wealthiest quintile but only about 53% for the two poorest quintiles. Some provinces had over three-fold differences in infant mortality rates and health infrastructure compared to others. Health expenditure in Indonesia remained below the average for low- and middle-income countries, accounting for only 3.1% of GDP in 2012. Out-of-pocket health expenses were 60%, further reflecting inequalities in healthcare access (Indonesia (BPS) 2013).

In response to these challenges, the Indonesian government increased public health expenditure (by 313% from 2010 to 2017, see Table 1) and implemented a series of health reforms, including the introduction of the Jaminan Kesehatan Nasional (JKN) in 2014. Life expectancy rose from 63 years in 1990 to 70 in 2017, and U5MR fell from 52 deaths per 1000 live births in 2000 to 25 in 2017 (World Bank Open Data 2024). Previous research by Moler-Zapata et al. (2022) documented the causal effect of improving U5MR by 0.38% on average following a 1% increase in public health expenditure over 2004 to 2012. We updated their analysis over 2010 to 2017 and did not find significant causal effects (Supplementary Table A6). However, the impact of increased public health expenditure on health inequalities remains an important area of study.

**Table 1:** Descriptive statistics.

Variables	2010	2011	2012	2013	2014	2015	2016	2017	Mean
Under-5 mortality rate (per 1000)	46.19	36.39	47.78	46.12	39.71	34.79	40.25	37.69	40.83
	(25.06)	(21.24)	(50.84)	(26.74)	(30.47)	(35.93)	(36.57)	(44.06)	(33.78)
U5MR CI	−0.08	−0.07	−0.13	−0.19	−0.06	0.00	−0.09	0.01	−0.072
	(0.26)	(0.29)	(0.27)	(0.23)	(0.29)	(0.28)	(0.30)	(0.26)	(0.278)
Total public health expenditure pc (in IDR)	43 084	50 227	57 728	67 399	56 196	100 295	142 069	178 048	89 254
	(50 151)	(56 299)	(63 852)	(86 951)	(95 521)	(129 144	(164 409)	(211 953)	(130 958)
Total public health expenditure pc (in logs)	10.28	10.47	10.61	10.65	10.38	10.88	11.37	11.57	10.79
	(0.85)	(0.79)	(0.78)	(0.91)	(0.90)	(1.17)	(1.01)	(1.05)	(1.04)
Household expenditure pc (in IDR)	495 856	580 686	649 889	675 474	773 826	868 947	946 285	1 036 532	763 957
	(137 311)	(166 629)	(196 478)	(197 892)	(224 246)	(226 221)	(236 624)	(253 319)	(272 471)
Household expenditure pc (in logs)	13.09	13.24	13.35	13.39	13.53	13.65	13.74	13.83	13.49
	(0.22)	(0.23)	(0.24)	(0.23)	(0.23)	(0.21)	(0.21)	(0.21)	(0.33)
Poverty rate (in % of population)	0.13	0.13	0.12	0.11	0.11	0.11	0.11	0.11	0.11
	(0.06)	(0.05)	(0.05)	(0.05)	(0.04)	(0.04)	(0.04)	(0.04)	(0.04)
Literacy rate (in % of total population over 15)	0.93	0.93	0.93	0.94	0.95	0.95	0.95	0.96	0.95
	(0.05)	(0.05)	(0.05)	(0.05)	(0.04)	(0.04)	(0.04)	(0.04)	(0.04)
Access to electricity: total (in % of total household)	0.94	0.94	0.96	0.96	0.97	0.97	0.97	0.98	0.97
	(0.10)	(0.10)	(0.09)	(0.08)	(0.07)	(0.07)	(0.07)	(0.06)	(0.06)
Access to safe sanitation (in % of total household)	0.65	0.65	0.67	0.70	0.71	0.74	0.76	0.78	0.71
	(0.08)	(0.08)	(0.08)	(0.08)	(0.08)	(0.07)	(0.07)	(0.06)	(0.08)
Immunization coverage (% of children population)	0.95	0.95	0.95	0.95	0.96	0.93	0.93	0.93	0.95
	(0.04)	(0.03)	(0.03)	(0.03)	(0.03)	(0.04)	(0.04)	(0.04)	(0.03)
Total own-source revenue (in logs)	28.52	28.81	28.96	29.12	29.30	29.36	29.40	29.49	29.22
	(1.23)	(1.16)	(1.15)	(1.18)	(1.20)	(1.17)	(1.18)	(1.17)	(1.17)
Oil exports (in logs)	5.02	6.10	5.95	6.19	5.83	3.14	2.83	3.33	4.96
	(8.86)	(10.76)	(10.48)	(10.91)	(10.27)	(5.54)	(4.99)	(5.87)	(8.86)

Standard deviations in parentheses under means. All descriptive statistics are constructed by taking the province-level values and weighting them by province population except for household expenditure per capita, which is defined at the household level.

Evidence from Indonesia is particularly relevant given its decentralized health system and uneven distribution of public resources across regions. There appears to be limited literature specifically addressing how health expenditure affects health disparities across different socioeconomic groups and regions in Indonesia. Sparrow et al. (2013) highlight how the expansion of the Jamkesmas programme improved service utilization but note persistent disparities in outcomes. Similarly, Rieger and Wagner (2015) show that while health insurance coverage in Indonesia increased, it did not translate into equitable improvements in child health.

The methodological framework proposed in this study can be applied to similar analyses globally according to the relevant local equity concern and associated priority population groups. Similarly, the approach can be applied to different mortality and morbidity indicators, and not only to whole health systems but also to evaluate a specific health policy intervention. The wide applicability of this methodological framework can contribute to more targeted and effective strategies for achieving health equity in line with the goals of Universal Health Coverage.

## Materials and methods

### Data

The data sources were previously used by Moler-Zapata et al. (2022) to estimate the marginal productivity of public health expenditure over the period 2004–12. For this study, we update the dataset through 2017. The main health outcome variable is the mortality rate for children under 5 years of age, defined as the probability for a given child of dying between birth and the age of 5 years. It is calculated using 5 years retrospective birth histories of women 15–49 years old, including birth date (month and year) for each child, sex of each child, whether each child is alive or deceased, current age of living children and age at death for deceased children. This information was collected by the Indonesian Demographic Health Survey (IDHS) in 2012 and 2017 (Indonesia 2008, Indonesia (BPS) 2013). Using these retrospective birth histories, we calculate yearly U5MR for each province from 2010 to 2017 using the Stata module SYNCMRATES (Masset 2016), which replicates the mortality rates reported in DHS studies using a synthetic cohort probability method (Elkasabi 2019). This approach allows us to generate annual U5MR estimates for up to five years preceding each survey: the 2012 IDHS provides estimates for 2010–12, while the 2017 IDHS provides estimates for 2013–17. The 2012 IDHS surveyed 83 650 individuals and the 2017 IDHS surveyed 184 090 individuals, with an average provincial sample size of 5382 (SD = 1337) and 5414 (SD = 615), respectively. All analyses were conducted using the DHS-provided survey weights to ensure representativeness and to account for the complex survey design.

The socioeconomic level variable used is a wealth index calculated using principal component analysis of household assets at the national level, available in the IDHS for 2012 and 2017 (Indonesia 2008, Indonesia (BPS) 2013, BKKBN et al., 2018). We divided households into quintiles at the provincial level based on the distribution of these national wealth index scores within each province. This approach maintains the national standardization of asset weights from the DHS methodology while ensuring that each province has households distributed across all five wealth quintiles.

The treatment variable is the total public health expenditure per capita aggregated at the provincial level per year from 2010 to 2017. These data are reported by the Indonesian Ministry of Finance (*Data Realisasi APBD Fungsi Pendidikan dan Kesehatan Provinsi 2010–2018*), as the local government health budget. It aggregates the realized health budgets of each province and is explicitly classified by government function. This dataset is based on the official APBD (*Anggaran Pendapatan dan Belanja Daerah*) system, i.e. the consolidated subnational budget execution framework, and therefore reflects harmonized administrative data that are directly comparable across provinces and over time.

We control for factors that could potentially confound the relationship between U5MR and public health expenditure. These covariates were obtained from the Indo-DAPOER database (World Bank, 2024), which harmonizes data from the *Survei Sosial Ekonomi Nasional (SUSENAS)* conducted annually by *Badan Pusat Statistik* (*BPS*). We control for total household expenditure, given that higher household purchasing power affects health outcomes by enabling access to improved nutrition, better quality services, and housing. We include the annual rate of relative poverty, given its influence on both U5MR and shaping decisions of health expenditure (Utomo et al. 2011). Further covariates related to critical determinants of child mortality, namely sanitation rates, given the prevalence of diseases related to diarrhoea (Komarulzaman et al. 2017); and immunization rates (Agustina et al. 2019) were included. We also accounted for provincial electrification rates and literacy rates among individuals aged 15 years and older.

#### Sample description

Table 1 presents descriptive statistics of our sample for 2010 to 2017 and the overall mean for the entire period. The U5MR, expressed as deaths per 1000 live births, was 40.83 for the analysed years, and followed a downward trend with a reduction of about 18% from 2010 to 2017. The U5MR CI measures the socioeconomic-related inequality in U5MR. A negative value indicates that U5MR is concentrated among the poor. The mean CI was −0.07, indicating that under-five mortality was disproportionally concentrated among poorer households throughout the study period. Values remained negative in most years, suggesting persistent socioeconomic inequality in child mortality, and the positive value in 2017 (0.01) is small relative to its standard error, limiting any substantive interpretation. The total public health expenditure per capita IDR shows a clear upward trend, going from 43 084 IDR in 2010 to 178 048 IDR in 2017, a substantial growth of about 313% over the period. Increases were particularly marked between 2014 and 2015, coinciding with the introduction of *JKN* (2014) (Agustina et al. 2019). JKN was launched in 2014 as a unified national insurance programme, whose introduction was heterogenous, implemented at different pace across district and provinces. Other indicators related to health, economic stability, infrastructure, and public services show a positive trend suggesting development progress over the period studied.

**Figure 1:**
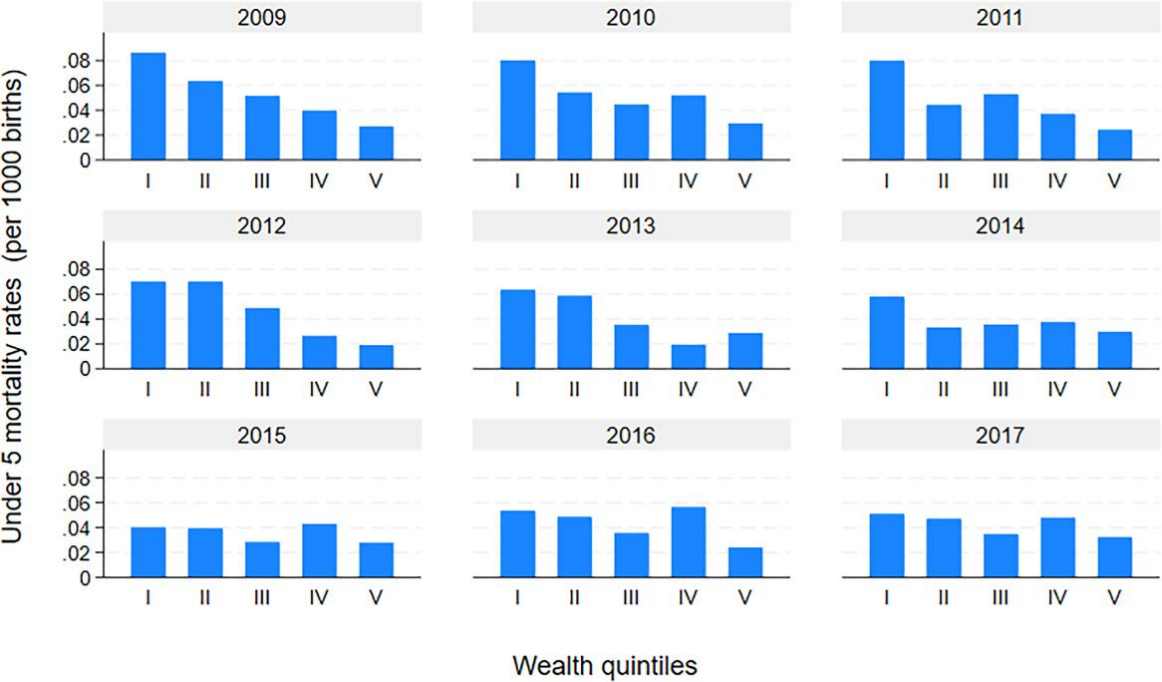
Under-five mortality rates (U5MR) by household wealth quintile, Indonesia 2010–17. Under-5 mortality rates (U5MR, per 1000 live births) are shown by household wealth quintile (I = poorest, V = richest) for Indonesian provinces. Estimates are derived from retrospective birth histories in the 2012 and 2017 Indonesian Demographic and Health Surveys (IDHS) using a synthetic cohort probability method (SYNCMRATES). Wealth quintiles are constructed at the provincial level based on the DHS asset index. All estimates apply survey weights.

Figure 1 displays U5MR by household wealth quintile. Using retrospective birth histories from the 2012 and 2017 IDHS, we estimated U5MR for each of the five province-specific wealth quintiles, covering 2010–12 and 2013–17 respectively. This produced quintile-specific mortality rates for each province-year. The results reveal a persistent socioeconomic gradient in child survival: poorer quintiles consistently face higher mortality rates compared to wealthier quintiles. Although U5MR declined gradually over time, the relative disadvantage of poorer quintiles remained, indicating that mortality reductions were not equitably distributed across the wealth spectrum.

#### Inequality measure

We measured socioeconomic-related inequality in UM5R using the CI, following the formulation of Wagstaff et al. (1991) and the grouped-data approach of O’Donnell et al. (2008), as our health variable of interest is only observed at the group level. The CI summarizes how under-five mortality is distributed across socioeconomic groups. A value of zero indicates that mortality is equally distributed across the population. Negative values indicate that mortality is disproportionately concentrated among poorer households, while positive values indicate that mortality is disproportionately concentrated among wealthier households. Values closer to zero indicate a more equal distribution of mortality across wealth groups, whereas larger absolute values indicate greater inequality.

U5MR was estimated from retrospective birth histories in the 2012 and 2017 IDHS. To construct the panel, we calculated U5MR for the years preceding each survey wave: the 2012 IDHS provided annual rates for 2010–12, and the 2017 IDHS provided annual rates for 2013–17. Within each survey wave, households were ranked into quintiles using the DHS asset-based wealth index, and province-specific U5MR was calculated separately for each quintile. This yielded five quintile-specific mortality rates per province-year, expressed as deaths per 1000 live births.

For each province-year, we calculated the CI using quintile-specific mortality rates and population shares:


(1) $$CI=\frac{2}{\mu }\overset{5}{\underset{k=1}{\sum }}\;y_{k}p_{k}R_{k}-1$$

where *y_k_* is the U5MR for wealth quintile *k*, $p_{k}$ is the population share of quintile *k*, *μ* is the province-year mean U5MR, and *R_k_* is the relative rank of each quantile *k* in the wealth distribution.

We employed the CI because it provides a single summary measure of the socioeconomic gradient of under-five mortality and is widely used in health equity research (Wagstaff et al. 1991, O’Donnell et al. 2008). While alternative measures such as the slope index of inequality (SII), absolute concentration indices, or stochastic dominance tests provide complementary perspectives, we focused on the CI given our aim of assessing relative inequality trends within provinces over time. Because U5MR is a continuous rate measure rather than a bounded binary variable, the standard CI is appropriate. However, to address concerns about bounded outcomes, we additionally report Erreygers-corrected CIs as a robustness check (Supplementary Table A3), which produced similar results.

**Figure 2:**
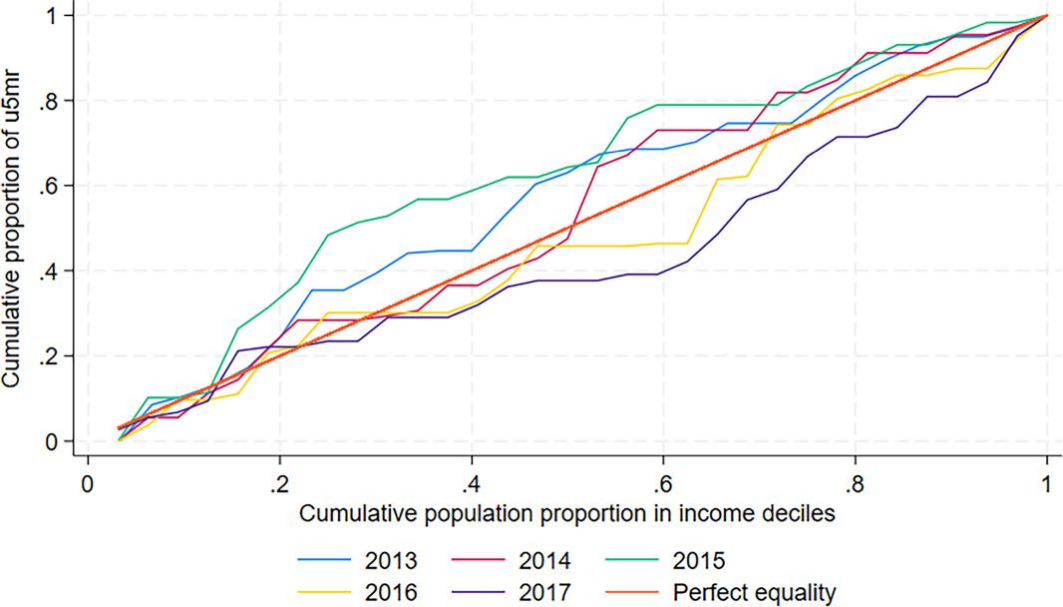
Concentration curves of U5MR. Concentration curves plot the cumulative proportion of under-5 mortality (U5MR) against the cumulative proportion of the population ranked by household wealth (from poorest to richest). The 45° line represents perfect equality. Curves lying above (below) this line indicate that mortality is disproportionately concentrated among poorer (richer) households.

Figure 2 illustrates the concentration curve underlying the CI. The curve plots the cumulative share of U5MR against the cumulative proportion of the population ranked from poorest to richest. A curve above the 45° line of equality indicates that mortality is concentrated among poorer households, while a curve below the line indicates the opposite. If the concentration curve coincides with the 45° line, the CI is zero, which implies perfect equality.

If the concentration curve crosses the line of equality, the direction of inequality differs across the wealth distribution. In such cases, the CI may be close to zero because inequalities favouring poorer households in some segments are offset by inequalities favouring wealthier households in others. Therefore, a CI close to zero does not necessarily imply the absence of inequality.

The CI is derived from the concentration curve as twice the area between the curve and the 45° equality line. In practical terms, the index converts the information contained in the entire curve into a single numerical summary of socioeconomic inequality in under-five mortality.

### Empirical strategy

#### Within-province inequality

This section estimates the impact of public health expenditure on socioeconomic inequality in U5MR. We define the relationship with a dynamic panel distributed-lag model following Gravelle and Backhouse (1987) and Moler-Zapata et al. (2022):


(2) $$c_{i,t}=\alpha_{t}+\lambda c_{i,t-1}+\beta_{0}d_{i,t}+\delta x_{i,t}+\gamma_{i}+\varepsilon_{i,t}$$

where *c_i,t_* represents the CI for each province *i* in year *t*, *x_i,t_* denotes a set of province-level covariates, *γ_i_* is the intercept for each province, and, *α_t_* is the year fixed effects term. The coefficient *λ* of the lagged dependent variable *c_i,t_*_−1_ captures the persistent effects of past expenditures. The coefficient of interest is *β*_0_, which is interpreted as the semi-elasticity of socioeconomic-related inequality. Specifically, a 1% increase in public health expenditures is associated with a *β*_0_/100 unit change in the CI.

There may be unobserved provincial characteristics that simultaneously affect both health expenditure and inequality potentially leading to endogeneity bias. These include governance quality, provincial health system efficiency, cultural or behavioural factors affecting service use, and informal private healthcare markets. Although we include proxies such as literacy, poverty, and electrification, not all drivers of inequality are observable in our dataset. If some of these factors are not fully captured by the control variables, and their changes are correlated with province-specific changes in health expenditure, the estimated effect of public spending will be subject to omitted variable bias.

These concerns informed our use of system Generalized Method of Moments (system-GMM) developed in Arellano and Bond (1991), Arellano and Bover (1995), Blundell and Bond (1998). The system-GMM is a technique for estimating parameters in panel data models, particularly when dealing with endogeneity, serial correlation, and unobserved heterogeneity relying on lagged variables of the explanatory variables as instrumental variables for them. To mitigate risks of instrument proliferation in the relatively small *T* (8 years) and moderate *N* (33 provinces) setting, we collapsed instruments following Roodman (2009) and report results using the finite-sample corrected two-step standard errors of Windmeijer (2005).

The method is robust to serial correlation which is likely to be present in our panel dataset as health inequality is likely to exhibit persistence over time. The system-GMM approach accounts for the fact that past inequalities influence current inequalities handling the correlation between the lagged dependent variable and the error term. Moreover, the distributed-lag specification of the method captures the delayed impact of health expenditure of inequality as it is unlikely for this effect to be immediate due to policy implementation, infrastructure development or behavioural changes. The system-GMM method, by using the lagged values of the endogenous regressors, helps to address the endogeneity concern of reverse causality of public health expenditure where it is possible that inequality levels may affect health spending even after controlling for province fixed effects.

In addition to the internal instruments provided by the system-GMM framework, we use external instruments that are correlated with public health expenditure but uncorrelated with unobserved determinants of U5MR inequality. Specifically, the log of provincial own-source revenue, as higher local revenue is associated with higher government capacity to fund healthcare but should not directly influence health inequality other than its effect on expenditure. The second instrument is provincial oil and gas exports: resource wealth influences government fiscal capacity affecting health budgets but not directly U5MR inequalities at the provincial level. Both are well-established proxies for provincial fiscal capacity in the Indonesian decentralization context (Bahl and Martinez-Vazquez 2006, Fadliya and McLeod 2010, Lewis 2010) and are therefore strong predictors of provincial fiscal capacity and, consequently, of health expenditure levels. That is, while they may influence overall levels of expenditure, they do not plausibly determine whether reductions in mortality accrue disproportionately to richer or poorer quintiles. Accordingly, these instruments are valid for identifying the causal effect of expenditure on U5MR inequality. These variables therefore satisfy the dual requirements of instrument relevance and exogeneity. Weak instrument diagnostics were assessed by examining Hansen test *P* values and the AR(2) test for serial correlation, both of which supported the validity of our instrument set.

#### Between-province inequality

We examine the effect of public health expenditure on the entire distribution of U5MR on the Indonesia panel dataset used earlier using the instrumental quantile regression method proposed by Chernozhukov and Hansen (2006). The method allows for estimation of causal effects across different quantiles of the conditional distribution of the outcome variable, in this case U5MR, rather than just reporting average effects. This allows us to examine heterogeneity in treatment effects across regions, i.e. whether public health expenditure reduce mortality more in high-mortality provinces than in low-mortality provinces.

The technique extends traditional quantile regression by addressing endogeneity through instrumental variables. We account for the panel structure of our data by including a lagged outcome as a regressor and lagged endogenous regressors as instrumental variables. This method addresses the potential endogeneity problem arising from potential reverse causality and omitted variable bias between public health expenditure and U5MR. Specifically, we account for the possibility that higher U5MR may lead to increased government health spending; or that unobserved factors may drive both expenditure and U5MR, even after controlling for province fixed effects. The IV-QTE approach corrects for this by using instrumental variables that are correlated with public health expenditure but uncorrelated with unobserved factors affecting mortality.

We define a model for the under 5 mortality rate *Y* with an endogenous treatment variable *D* for province-level public health expenditure and extend it to a dynamic panel specification by incorporating lagged dependent variables and additional covariates:


(3) $$Q_{Y_{it}}(\tau |Y_{i,t-1},D_{it},X_{it},\alpha_{i})=\rho (\tau )Y_{i,t-1}+D_{it}^{\prime }\beta (\tau )+X_{it}^{\prime }\delta (\tau )+\alpha_{i}(\tau )$$

where *Q_y_* is the *τ*th conditional quantile function of the U5MR, *Y_i,t_*_−1_ is the lagged outcome; *X_i,t_* are province-level covariates; *β(τ)* is the coefficient of interest, capturing the effect of public health expenditures on U5MR for the *τ*th quantile; and *α_i_(τ)* represents individual-specific effects that may vary across quantiles.

## Results

### Within-province inequality

Table 2 shows the dynamic panel System-GMM estimates of the causal effect of public health expenditures on socioeconomic-related inequality in U5MR, measured by the CI. The estimated coefficients are consistently negative across specifications, ranging from −0.3 to −0.19. Our preferred specification (column 1) yields a coefficient of −0.29 (*P* < .05), implying a 1% increase in provincial health expenditure per capita causes the CI of U5MR to decline by −0.0029 points, a 4% increase in relative inequality. Because negative values of the CI indicate that mortality is concentrated among poorer households, this result implies that additional spending was associated with a widening of inequality. In practical terms, mortality reductions appear to have benefited healthier households more than poorer households.

**Table 2:** Effect of public health expenditure on under-5 mortality socioeconomic-related inequality.

	(1)	(2)	(3)	(4)
Variables	CI	CI	CI	CI
Total public health expenditure per capita	−0.292**	−0.279*	−0.300**	−0.190
	(0.118)	(0.160)	(0.134)	(0.286)
Household expenditure per capita	0.994*	0.960	0.996*	0.657
	(0.516)	(0.607)	(0.577)	(0.993)
Poverty rate	2.228	1.923	2.205	1.298
	(2.200)	(2.551)	(2.248)	(3.266)
Literacy rate	−1.404	−1.489	−1.660	−0.848
	(1.311)	(1.305)	(1.530)	(2.499)
Electricity rate	−1.398	−1.431	−1.507*	−1.469*
	(0.857)	(0.973)	(0.873)	(0.786)
Sanitation rate	−0.394	−0.291	−0.424	−0.535
	(0.460)	(0.510)	(0.524)	(0.529)
Children immunization	1.432	1.482	1.517	1.605
	(2.429)	(2.546)	(2.289)	(1.720)
Lagged CI	0.347	0.348	0.262	0.176
	(0.240)	(0.258)	(0.324)	(0.340)
Observations	231	231	231	231
Instruments Log of own-source revenue	✓	✓		
Oil exports	✓		✓	
Lagged values of the endogenous variables	✓	✓	✓	✓
Total number of instruments	31	30	30	29
Number of provinces	32	32	32	32
Arellano–bond AR (1) test in differences (*P* value)	0.015	0.017	0.051	0.105
Arellano–bond AR (2) test in differences (*P* value)	0.848	0.847	0.971	0.885
Hansen J-test (*P* value)	0.408	0.372	0.344	0.217

This table reports estimates of Eq. (2) for the period 2010–17 using a panel of 32 Indonesian provinces. The dependent variable is the CI of under-5 mortality, where more negative values indicate a greater concentration of mortality among poorer households. All specifications are estimated using two-step system-GMM with the first-difference transformation and include year fixed effects. Lagged values of the endogenous variables are used as internal instruments, while columns (1)–(4) differ in the inclusion of external instruments (log of own-source revenue and oil exports), as indicated by the checkmarks. Public health expenditure per capita is included in logarithms. Standard errors are Windmeijer-corrected for finite samples and reported in parentheses. The Arellano–Bond tests report *P* values for first- and second-order serial correlation in first differences. The Hansen J-test reports the *P* value for the null hypothesis that the instrument set is valid. The total number of instruments is reported for each specification. ***P < .01, **P < .05, *P < .1.

Put differently, the dynamic panel approach indicates that inequality does not simply reflect cross-sectional differences across provinces, but that the trajectory of inequity within provinces is linked to their fiscal choices. The estimated coefficient on health expenditure should therefore be interpreted as the marginal effect of expenditure increases on inequality trajectories, rather than on static differences.

Diagnostic tests support the validity of our identification strategy. The second-order Arellano–Bond autocorrelation tests indicate no evidence of serial correlation in the error term, suggesting that the lags are valid instruments. Similarly, the results of the Hansen J-test do not reject the null hypothesis of over identifying restrictions, implying that our additional instruments (provincial own-source revenue and oil and gas exports) are exogenous and valid to estimate the effect of public health expenditures on socioeconomic-related inequality in U5MR.

To assess whether the equity effects of expenditure varied by settlement type, we disaggregated the analysis by urban and rural strata. The findings suggest that the pro-rich bias of public health spending was more pronounced in rural populations, where additional resources appeared to translate into greater relative gains for wealthier households. By contrast, in urban populations, the estimated effects had the opposite sign. This result is consistent with the idea that rural groups, facing limited access to health infrastructure and services, are less able to benefit equitably from new spending, allowing relatively better-off households to capture a disproportionate share of the gains, while urban populations are better positioned to experience more equitable improvements. At the same time, these results should be interpreted cautiously: disaggregating to province–quintile–stratum levels substantially reduces sample sizes, rural estimates are imprecise, and some specifications fail standard diagnostic tests. Together, these caveats highlight the need for richer sub-provincial data to more definitively assess urban–rural differences in the distributional impact of health spending.

While the estimated effect of public health expenditure on inequality is stable across specifications, some variation in the underlying data across years likely reflects differences in mortality trajectories and limited sample precision at the province–quintile level. These limitations do not affect the main conclusion that increases in public health expenditure are associated with worsening relative inequality in U5MR.

### Between-province inequality

**Figure 3:**
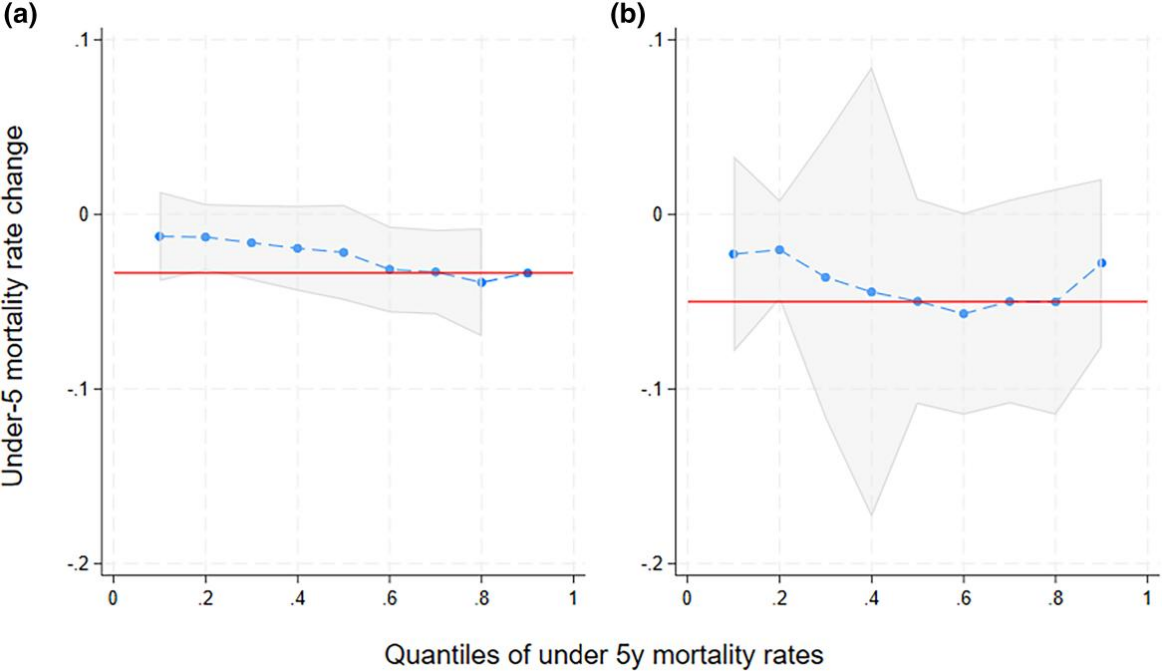
Quantile treatment effect of public health expenditure on under-5 mortality rates. Figure 3 reports instrumental variable quantile treatment effect (IV-QTE) estimates of the effect of public health expenditure on under-5 mortality rates (U5MR) across the conditional distribution of U5MR. Panel (a) presents estimates without additional covariates, while Panel (b) includes controls for household expenditure, poverty, literacy, sanitation, and electrification at the provincial level. The dashed line shows the estimated QTE at each quantile, while the solid horizontal line represents the corresponding mean (average) effect. Shaded areas indicate 95% confidence intervals. U5MR change refers to the estimated marginal effect of an increase in public health expenditure per capita on U5MR (i.e. the change in the under-5 mortality rate associated with a one-unit increase in expenditure).

Figure 3 presents the quantile treatment effects of public health expenditures on U5MR. Panel A shows a negative effect for all points of the distribution; however, it is not statistically significant. The width of the confidence intervals indicates great uncertainty in the estimates, likely due to the sample size limitation. The results do not show heterogeneous effect across the distribution of U5MR. Our estimates presented in Panel B control for total household expenditure, poverty, literacy, sanitation, and electrification rates at the provincial level. There is some heterogeneity in the effect as public health expenditure seems to have a larger effect in the quantiles at the lower end of U5MR. This suggests that provinces with lower mortality rates experience larger reductions in U5MR from increased health expenditure. However, the effect is not statistically different from zero.

Notably, conditional quantiles represent effects of public health expenditure on U5MR given a specific set of control variables. These differ from unconditional quantiles, which capture effects across the entire population distribution without conditioning on covariates. The implication for interpretation is that the stronger effect of public health expenditure observed at lower quantiles of U5MR does not imply that provinces with lower mortality benefit more in absolute terms. Rather it indicates that, within provinces with otherwise similar characteristics, those starting with relative lower mortality exhibit a larger proportional response to changes in health expenditure, compared to provinces at higher mortality levels.

In addition, we estimated the average effect of public health expenditure on under-five mortality rates across four System-GMM specifications. The coefficient on total public health expenditure per capita is consistently negative across all models, suggesting that higher spending is associated with reductions in U5MR. However, the estimates are small in magnitude and not statistically significant in any specification, indicating no robust evidence of an average effect of public health expenditure on mortality outcomes.

## Discussion

This study extends existing research by applying methodological approaches to assess how public health expenditure affects inequality in health outcomes. Estimating these heterogeneous impacts is critical for designing policies that equitably improve population health, a central UHC objective. Specifically, our analysis sheds light on whether increased government health spending in Indonesia between 2010 and 2017 translated into reductions in under-five mortality rate inequality between poorer and wealthier households, and between provinces with differing levels of U5MR.

We evaluated the impact of public health expenditure on health inequality using two approaches. First, we assessed how changes in public health expenditure affected the CI of under-five mortality rate within provinces. This metric summarizes how child mortality is distributed across socioeconomic groups. Second, we examined whether public health expenditure had differential effects across provinces with varying baseline mortality burdens, using instrumental quantile regression. Together, these analyses allowed us to investigate both within- and between-province inequalities.

Our findings show that public health spending was associated with a worsening of inequality in under-five mortality across socioeconomic groups. Specifically, increased expenditure was linked with a more unequal distribution of under-five mortality across wealthier groups. Mortality reductions were larger among wealthier households than among poorer households, leading to a widening of socioeconomic inequality. This may occur if health services funded through increased spending were more accessible to better-off households, either due to geographic location, health system infrastructure, or informational advantages.

We found no statistically significant evidence that health expenditure had differential effects across the distribution of provincial U5MR. While point estimates suggested that provinces with lower U5MR experienced somewhat greater marginal gains from spending, the confidence intervals were wide, and the effects were not statistically significant. This suggests limited responsiveness of spending allocation to regional health needs during the study period.

In addition, public health expenditure is associated with small negative (mortality-reducing) coefficients, but these are not statistically significant. This suggests no robust average effect on U5MR, consistent with our main findings that increased spending does not necessarily translate into improved or more equitable health outcomes.

These findings align with recent evidence indicating that public health spending in Indonesia is subject to strong geographic, urban–rural, and wealth-based disparities. For example, Haemmerli et al. (2021) show that primary health care facilities in poorer, rural, or otherwise disadvantaged communities have lower readiness scores and lower provider knowledge, which could limit how well increased funding translates into improved outcomes. Laksono et al. (2023) find that hospital utilization in 2018 remains substantially higher in urban than rural provinces, even after adjusting for insurance status and travel time, indicating persistent access barriers. Mulyanto et al. (2019) document that income-related inequalities in healthcare utilization have declined for some services but increased again for others by 2014, suggesting that improvements are not uniform. These findings support our hypothesis that infrastructure, service quality, and utilization gaps are plausible mechanisms through which increased expenditure may fail to reduce, or may even increase, inequalities in U5MR. Incorporating data on facility readiness, geographic access to care, and utilization rates in future work would help in empirically testing these mechanisms.

Although our empirical analysis ends in 2017, the findings remain highly relevant for Indonesia’s ongoing reforms. The persistence of pro-rich bias in the distribution of child mortality gains underscores the importance of designing financing mechanisms that explicitly target vulnerable groups. Under JKN, the shift towards national social health insurance and capitation-based primary care financing should help mitigate inequities if capitation formulas are risk-adjusted and if *Dana Alokasi Khusus* (DAK) transfers incorporate explicit need-based criteria. However, without equity-sensitive allocation rules, the inverse care law identified in our study, which states that wealthier groups benefit disproportionately from increased health expenditure, may persist or even be reinforced under insurance expansion. Our analysis using 2010–17, concludes that reforms like JKN did not narrow inequalities in under-five mortality.

This finding aligns with broader international evidence suggesting that the progressive realization of UHC requires attention not only to reduce levels but also to allocation mechanisms (Norheim 2016, Ahmed et al. 2022). Traditional benefit incidence analyses in LMICs have often shown that wealthier groups capture a disproportionate share of public health subsidies, particularly for hospital services (O’Donnell et al. 2008, Akazili et al. 2012). Our approach extends this tradition by estimating the causal effect of expenditure on inequality in outcomes rather than service use, thereby providing complementary insights for policymakers. Similarly, recent work on equity-weighted cost-effectiveness analysis (Asaria et al. 2016, Cookson et al. 2017) emphasizes that the social value of health interventions depends not only on efficiency but also on distributional consequences. The finding that additional spending can worsen relative inequalities in U5MR reinforces the need for such distribution-sensitive frameworks when evaluating fiscal priorities. Evidence from other LMICs shows that expansions in public spending or insurance coverage can leave relative inequalities unchanged or even exacerbate them—for example, Mexico’s *Seguro Popular* initially increased utilization more among wealthier groups (Gakidou et al. 2006), while India’s *Rashtriya Swasthya Bima Yojana* disproportionately benefited higher-income households in some states (Karan et al. 2017). Situating Indonesia’s experience within this broader evidence base highlights a generalizable lesson: more spending alone is insufficient to close equity gaps without deliberate targeting of disadvantaged groups.

### Conclusions

This study examined the relationship between public health expenditure and socioeconomic inequality in under-five mortality (U5MR) in Indonesia between 2010 and 2017. The results indicate that increases in public expenditure in this period did not translate into reductions in under-five mortality socioeconomic inequality; instead, the gains appear to have been captured disproportionally by better-off households, likely due to differential ability to access or benefit from publicly funded services. The absence of significant effects across provinces with varying mortality burdens further suggests that allocation of health resources was not well-targeted towards areas of greatest need.

### Policy implications

The findings of this study have direct implications for the design of Indonesia’s health financing system and for low- and middle-income countries pursuing Universal Health Coverage. The persistence of the inverse care law suggests that merely raising the level of public spending is insufficient to reduce inequalities in child mortality. Instead, equity-oriented health financing strategies should prioritize targeted investments in primary and preventive care, particularly in rural and underserved areas, while ensuring that services are physically and socially accessible to poorer populations. This perspective aligns with the principle of progressive universalism, which emphasizes directing incremental public health investments towards populations with the greatest unmet health needs to ensure that progress towards Universal Health Coverage is both inclusive and equitable (Gwatkin and Ergo 2011, Hanson et al. 2022).

At the national policy level, our findings highlight the potential value of including stronger equity considerations within Indonesia’s fiscal and insurance mechanisms. For instance, future work could examine whether formula-based allocations under the DAK or adjustments to JKN’s capitation payments that account for population health needs, poverty, and geographic access might enhance the equity of public resource distribution. Likewise, exploring how different types of local health investments, such as primary healthcare infrastructure, community outreach, or transport support, affect service use across socioeconomic groups could help identify the channels through which public expenditure influences inequality.

Internationally, these issues resonate with discussions on integrating distributional considerations into economic evaluations and public budgeting for health (Claxton et al. 2015, Ochalek et al. 2018). While the expansion of health insurance coverage under JKN marks significant progress (Agustina et al. 2019), its implications for outcome equity remain to be fully assessed. Comparative evidence from other contexts also points to the relevance of policies that enhance affordability, accessibility, and community-level service delivery (Ensor et al. 2008, Quayyum et al. 2010, Syed et al. 2013), though the applicability of such measures in Indonesia warrants empirical investigation.

### Limitations and future directions

We acknowledge a few limitations in our methods and data. First, the analysis relies on provincial-level expenditure and inequality measures, which may mask within-province heterogeneity. Aggregation bias could attenuate measure inequalities if disparities are sharper at the district or community level, suggesting that our estimates may understate the true extent of socioeconomic gaps. Second, the use of retrospective birth histories from the IDHS to construct annual under-five mortality rates may introduce recall bias, potentially affecting U5MR accuracy. Such error is likely to be non-systematic and would bias results towards zero, making our estimates conservative. Third, inequality was measured using the group-data CI method, based on quintile-specific U5MR calculated at the province level. This approach is widely applied in health equity studies (O’Donnell et al. 2008), but it may smooth within-quintile variation and attenuate measured inequality relative to microdata-based methods. Moreover, the weighting scheme reflects the distribution of births across quintiles, which appropriately captures the population at risk but may not fully account for other demographic dynamics such as fertility differentials.

Fourth, our analysis relies on only two survey waves (2012 and 2017) to construct an eight-year panel (2010–17). While this provides valuable temporal coverage, it assumes comparability of retrospective reports across waves and stable socioeconomic rankings between rankings.

The fifth limitation concerns statistical power. Although the IDHS provides large national samples, disaggregating the data to construct annual U5MR by province and wealth quintile substantially reduces the effective sample size. This limited precision at the disaggregated level may increase measurement variability in the inequality estimates and raise the risk of type II error, meaning that some true effects could go undetected.

Although all variables are available prior to 2010, the analysis is limited to 2010–17 due to inconsistencies in the public health expenditure data, which come from two non-comparable series (2002–12 and 2010–19). We rely exclusively on the more recent series and therefore limit the analysis to its period of coverage. The study should be viewed as a proof of concept, with a methodological framework that can be applied to harmonized data as they become available.

Despite these caveats, we undertook several robustness checks, including alternative specifications, and found consistent results. Moreover, diagnostic tests support the validity of our identification strategy. Taken together, these considerations suggest that, if anything, our results are conservative and the true equity impact of expenditure may be even more pronounced. Future research with district-level panel data, direct measures of household health expenditure, and microdata-based concentration indices would help address these limitations more fully.

To build on this work, future analyses would benefit from enhanced data availability and granularity. Extending the analysis period of this study would allow researchers to assess the long-term equity effects of the country’s major health financing reforms. Access to district-level panel data would improve the precision of estimates and provide a clearer understanding of local variation in health outcomes and spending impacts. Broadening the scope of health outcomes to include adult mortality, non-communicable diseases, and service utilization measures would provide a more complete picture of equity impacts. Future work could usefully compare our results using relative CI, to absolute measures (e.g. SII) or dominance approaches to provide a more comprehensive equity assessment. Finally, incorporating detailed data on out-of-pocket health expenditures and more nuanced measures of socioeconomic status (e.g. housing quality, social networks, gender dynamics, and neighbourhood characteristics) would enable better identification of the structural determinants of health inequality. These data improvements would enable a more robust application of distribution-sensitive methods and support the design of targeted, equity-oriented health financing policies.

## Supplementary Material

czag081_Supplementary_Data

## Data Availability

The data underlying this article will be shared on reasonable request to the corresponding author.

## Reflexivity statement

We have prioritized equitable collaboration, centring local expertise throughout the research process and ensuring inclusive participation in study design, analysis, and authorship decisions. Our six-member author team reflects balance in gender (three women and three men), career stage (three senior and three junior researchers), and geographic background. Three authors are from Indonesia—the country studied—ensuring strong local representation. In total, four of six authors are from LMICs. Based in Indonesia (2), the UK (3), and the US (1), we bring diverse perspectives across contexts.
