# Supplementary material for: Does increasing overall health expenditure reduce inequality in under 5 mortality rates between provinces in Indonesia?
Source: Health Policy Plan. 2026 Jun 18;41(7):1242–54. doi: 10.1093/heapol/czag081 (PMC13481677; doi:10.1093/heapol/czag081)
Supplement: Supplementary file 1 [file czag081_supplementary_data.docx]

# Supplementary material for: Does increasing overall health expenditure reduce inequality in under 5 mortality rates between provinces in Indonesia?

# Appendix A1. Robustness checks

**Table A1: Effect of public health expenditure on under-5 mortality socioeconomic-related inequality (excluding sanitation rates)**

|  | (1) | (2) | (3) | (4) |
| --- | --- | --- | --- | --- |
| VARIABLES | CI | CI | CI | CI |
| Total public health expenditure per capita | -0.300** | -0.273* | -0.328** | -0.202 |
|  | (0.119) | (0.144) | (0.129) | (0.285) |
| Household expenditure per capita | 0.967* | 0.935 | 1.067* | 0.679 |
|  | (0.555) | (0.553) | (0.547) | (1.013) |
| Poverty rate | 2.245 | 2.103 | 2.566 | 1.633 |
|  | (2.724) | (2.465) | (2.495) | (3.386) |
| Literacy rate | -1.701 | -2.024 | -2.407 | -1.616 |
|  | (1.447) | (1.244) | (1.544) | (2.711) |
| Electricity rate | -1.356 | -1.417 | -1.399 | -1.413* |
|  | (0.820) | (0.844) | (0.826) | (0.722) |
| Children immunisation | 1.091 | 1.220 | 0.943 | 1.240 |
|  | (2.549) | (2.389) | (2.299) | (1.778) |
| Lagged concentration index | 0.333 | 0.240 | 0.324 | 0.136 |
|  | (0.289) | (0.280) | (0.312) | (0.331) |
| Observations | 231 | 231 | 231 | 231 |
| Instruments  Log of own-source revenue | ✓ | ✓ |  |  |
| Oil exports | ✓ |  | ✓ |  |
| Lagged values of the endogenous variables | ✓ | ✓ | ✓ | ✓ |
| Total number of instruments | 30 | 29 | 29 | 28 |
| Number of provinces | 32 | 32 | 32 | 32 |
| Arellano–Bond AR (1) test in differences (p value) | 0.027 | 0.038 | 0.033 | 0.105 |
| Arellano–Bond AR (2) test in differences (p value) | 0.877 | 0.978 | 0.889 | 0.786 |
| Hansen J-test (p value) | 0.387 | 0.373 | 0.382 | 0.243 |

This table reports estimates of Eq. (2) for the period 2010–2017 using a panel of 32 Indonesian provinces. This specification does not include sanitation rates as a control. The dependent variable is the concentration index (CI) of under-5 mortality. All specifications are estimated using two-step system GMM with the first-difference transformation and include year fixed effects. Lagged values of the endogenous variables are used as internal instruments, while columns (1)–(4) differ in the inclusion of external instruments (log of own-source revenue and oil exports), as indicated by the checkmarks. Public health expenditure per capita is included in logarithms. Standard errors are Windmeijer-corrected for finite samples and reported in parentheses. The Arellano–Bond tests report p-values for first- and second-order serial correlation in first differences. The Hansen J-test reports the p-value for the null hypothesis that the instrument set is valid. The total number of instruments is reported for each specification. *** p < 0.01, ** p < 0.05, * p < 0.1

**Table A2: Effect of public health expenditure on under-5 mortality socioeconomic-related inequality (adjusted by inflation, 2010, IDR)**

|  | (1) | (2) | (3) | (4) |
| --- | --- | --- | --- | --- |
| VARIABLES | CI | CI | CI | CI |
| Total public health expenditure per capita | -0.291** | -0.279* | -0.300** | -0.195 |
|  | (0.118) | (0.159) | (0.135) | (0.283) |
| Household expenditure per capita | 0.992* | 0.961 | 0.995* | 0.676 |
|  | (0.516) | (0.610) | (0.578) | (0.977) |
| Poverty rate | 0.022 | 0.019 | 0.022 | 0.014 |
|  | (0.022) | (0.026) | (0.022) | (0.032) |
| Literacy rate | -0.014 | -0.015 | -0.017 | -0.009 |
|  | (0.013) | (0.013) | (0.015) | (0.025) |
| Electricity rate | -0.014 | -0.014 | -0.015* | -0.015* |
|  | (0.009) | (0.010) | (0.009) | (0.008) |
| Sanitation rate | -0.004 | -0.003 | -0.004 | -0.005 |
|  | (0.005) | (0.005) | (0.005) | (0.005) |
| Children immunisation | 0.014 | 0.015 | 0.015 | 0.016 |
|  | (0.024) | (0.026) | (0.023) | (0.018) |
| Lagged concentration index | 0.351 | 0.350 | 0.266 | 0.183 |
|  | (0.243) | (0.259) | (0.327) | (0.342) |
| Observations | 231 | 231 | 231 | 231 |
| Instruments  Log of own-source revenue | ✓ | ✓ |  |  |
| Oil exports | ✓ |  | ✓ |  |
| Lagged values of the endogenous variables | ✓ | ✓ | ✓ | ✓ |
| Total number of instruments | 31 | 30 | 30 | 29 |
| Number of provinces | 32 | 32 | 32 | 32 |
| Arellano–Bond AR (1) test in differences (p value) | 0.015 | 0.017 | 0.051 | 0.103 |
| Arellano–Bond AR (2) test in differences (p value) | 0.843 | 0.843 | 0.964 | 0.897 |
| Hansen J-test (p value) | 0.404 | 0.369 | 0.342 | 0.219 |

This table reports estimates of Eq. (2) for the period 2010–2017 using a panel of 32 Indonesian provinces. Monetary values are expressed in constant IDR (2010). The dependent variable is the concentration index (CI) of under-5 mortality, where more negative values indicate a greater concentration of mortality among poorer households. All specifications are estimated using two-step system GMM with the first-difference transformation and include year fixed effects. Lagged values of the endogenous variables are used as internal instruments, while columns (1)–(4) differ in the inclusion of external instruments (log of own-source revenue and oil exports), as indicated by the checkmarks. Public health expenditure per capita is included in logarithms. Standard errors are Windmeijer-corrected for finite samples and reported in parentheses. The Arellano–Bond tests report p-values for first- and second-order serial correlation in first differences. The Hansen J-test reports the p-value for the null hypothesis that the instrument set is valid. The total number of instruments is reported for each specification.

*** p < 0.01, ** p < 0.05, * p < 0.1

**Table A3: Effect of public health expenditure on under-5 mortality socioeconomic-related inequality (Erreygers correction)**

|  | (1) | (2) | (3) | (4) |
| --- | --- | --- | --- | --- |
| VARIABLES | CI | CI | CI | CI |
| Total public health expenditure per capita | -0.027** | -0.027** | -0.024 | -0.016 |
|  | (0.013) | (0.013) | (0.017) | (0.030) |
| Household expenditure per capita | 0.101 | 0.100 | 0.097 | 0.068 |
|  | (0.070) | (0.078) | (0.076) | (0.106) |
| Poverty rate | 0.182 | 0.152 | 0.181 | 0.096 |
|  | (0.280) | (0.335) | (0.273) | (0.333) |
| Literacy rate | -0.159 | -0.157 | -0.150 | -0.087 |
|  | (0.222) | (0.231) | (0.234) | (0.269) |
| Electricity rate | -0.109 | -0.115 | -0.105 | -0.093 |
|  | (0.086) | (0.089) | (0.086) | (0.082) |
| Sanitation rate | -0.049 | -0.043 | -0.052 | -0.052 |
|  | (0.062) | (0.065) | (0.060) | (0.063) |
| Children immunisation | 0.267 | 0.275 | 0.293 | 0.286 |
|  | (0.303) | (0.320) | (0.280) | (0.243) |
| Lagged concentration index | 0.420** | 0.418** | 0.319 | 0.338 |
|  | (0.193) | (0.189) | (0.234) | (0.218) |
| Observations | 231 | 231 | 231 | 231 |
| Instruments  Log of own-source revenue | ✓ | ✓ |  |  |
| Oil exports | ✓ | ✓ | ✓ |  |
| Lagged values of the endogenous variables | ✓ | ✓ | ✓ | ✓ |
| Total number of instruments | 31 | 30 | 30 | 29 |
| Number of provinces | 32 | 32 | 32 | 32 |
| Arellano–Bond AR (1) test in differences (p value) | 0.016 | 0.017 | 0.039 | 0.034 |
| Arellano–Bond AR (2) test in differences (p value) | 0.930 | 0.939 | 0.902 | 0.982 |
| Hansen J-test (p value) | 0.237 | 0.206 | 0.153 | 0.120 |

This table reports estimates of Eq. (2) for the period 2010–2017 using a panel of 32 Indonesian provinces. The dependent variable is the concentration index (CI) of under-5 mortality adjusted with Erreygers correction, where more negative values indicate a greater concentration of mortality among poorer households. All specifications are estimated using two-step system GMM with the first-difference transformation and include year fixed effects. Lagged values of the endogenous variables are used as internal instruments, while columns (1)–(4) differ in the inclusion of external instruments (log of own-source revenue and oil exports), as indicated by the checkmarks. Public health expenditure per capita is included in logarithms. Standard errors are Windmeijer-corrected for finite samples and reported in parentheses. The Arellano–Bond tests report p-values for first- and second-order serial correlation in first differences. The Hansen J-test reports the p-value for the null hypothesis that the instrument set is valid. The total number of instruments is reported for each specification.

*** p < 0.01, ** p < 0.05, * p < 0.1

**Table A4: Effect of public health expenditure on under-5 mortality socioeconomic-related inequality (urban areas)**

|  | (1) | (2) | (3) | (4) |
| --- | --- | --- | --- | --- |
| VARIABLES | CI | CI | CI | CI |
| Total public health expenditure per capita | 0.049 | 0.108 | 0.067 | 0.149 |
|  | (0.101) | (0.127) | (0.106) | (0.112) |
| Household expenditure per capita | -0.475 | -0.670* | -0.581 | -0.799** |
|  | (0.328) | (0.392) | (0.370) | (0.387) |
| Poverty rate | -0.011 | -0.016 | -0.015 | -0.020 |
|  | (0.014) | (0.015) | (0.017) | (0.018) |
| Literacy rate | 0.011 | 0.015 | 0.013 | 0.016 |
|  | (0.012) | (0.012) | (0.012) | (0.013) |
| Electricity rate | 0.000 | 0.003 | 0.000 | 0.005 |
|  | (0.008) | (0.009) | (0.008) | (0.009) |
| Sanitation rate | -0.002 | -0.002 | -0.003 | -0.003 |
|  | (0.005) | (0.004) | (0.005) | (0.005) |
| Children immunisation | -0.005 | -0.006 | -0.004 | -0.008 |
|  | (0.012) | (0.013) | (0.012) | (0.014) |
| Lagged concentration index | 0.562* | 0.587** | 0.543* | 0.587** |
|  | (0.301) | (0.272) | (0.319) | (0.277) |
| Observations | 166 | 166 | 166 | 166 |
| Instruments  Log of own-source revenue | ✓ | ✓ |  |  |
| Oil exports | ✓ |  | ✓ |  |
| Lagged values of the endogenous variables | ✓ | ✓ | ✓ | ✓ |
| Total number of instruments | 31 | 30 | 30 | 29 |
| Number of provinces | 32 | 32 | 32 | 32 |
| Arellano–Bond AR (1) test in differences (p value) | 0.053 | 0.047 | 0.057 | 0.05 |
| Arellano–Bond AR (2) test in differences (p value) | 0.809 | 0.698 | 0.784 | 0.643 |
| Hansen J-test (p value) | 0.402 | 0.432 | 0.366 | 0.404 |

This table reports estimates of Eq. (2) for the period 2010–2017 using a panel of urban areas in 32 Indonesian provinces. The dependent variable is the concentration index (CI) of under-5 mortality, where more negative values indicate a greater concentration of mortality among poorer households. All specifications are estimated using two-step system GMM with the first-difference transformation and include year fixed effects. Lagged values of the endogenous variables are used as internal instruments, while columns (1)–(4) differ in the inclusion of external instruments (log of own-source revenue and oil exports), as indicated by the checkmarks. Public health expenditure per capita is included in logarithms. Standard errors are Windmeijer-corrected for finite samples and reported in parentheses. The Arellano–Bond tests report p-values for first- and second-order serial correlation in first differences. The Hansen J-test reports the p-value for the null hypothesis that the instrument set is valid. The total number of instruments is reported for each specification.

*** p < 0.01, ** p < 0.05, * p < 0.1

**Table A5: Effect of public health expenditure on under-5 mortality socioeconomic-related inequality (rural areas)**

|  | (1) | | (2) | | (3) | | (4) | |
| --- | --- | --- | --- | --- | --- | --- | --- | --- |
| VARIABLES | CI | | CI | | CI | | CI | |
| Total public health expenditure per capita | -0.018 | | -0.045 | | 0.002 | | -0.021 | |
|  | (0.130) | | (0.218) | | (0.135) | | (0.201) | |
| Household expenditure per capita | -0.107 | | -0.009 | | -0.233 | | -0.136 | |
|  | (0.516) | | (0.877) | | (0.574) | | (0.800) | |
| Poverty rate | 0.011 | | 0.016 | | 0.006 | | 0.011 | |
|  | (0.025) | | (0.046) | | (0.029) | | (0.043) | |
| Literacy rate | 0.002 | | 0.001 | | 0.005 | | 0.003 | |
|  | (0.012) | | (0.015) | | (0.013) | | (0.014) | |
| Electricity rate | -0.003 | | -0.004 | | -0.002 | | -0.003 | |
|  | (0.009) | | (0.014) | | (0.009) | | (0.012) | |
| Sanitation rate | 0.001 | | 0.001 | | -0.000 | | 0.000 | |
|  | (0.005) | | (0.006) | | (0.005) | | (0.007) | |
| Children immunisation | -0.005 | | -0.003 | | -0.004 | | -0.003 | |
|  | (0.016) | | (0.020) | | (0.016) | | (0.020) | |
| Lagged concentration index | 0.361*** | | 0.357** | | 0.393*** | | 0.383** | |
|  | (0.107) | | (0.152) | | (0.130) | | (0.156) | |
| Observations | 166 | | 166 | | 166 | | 166 | |
| Instruments  Log of own-source revenue | ✓ | | ✓ | |  | |  | |
| Oil exports | ✓ | |  | | ✓ | |  | |
| Lagged values of the endogenous variables | ✓ | | ✓ | | ✓ | | ✓ | |
| Total number of instruments | 28 | | 27 | | 27 | | 26 | |
| Number of provinces | 32 | | 32 | | 32 | | 32 | |
| Arellano–Bond AR (1) test in differences (p value) | 0.037 | 0.047 | | 0.039 | | 0.041 | |  |
| Arellano–Bond AR (2) test in differences (p value) | 0.857 | | 0.787 | | 0.946 | | 0.868 | |
| Hansen J-test (p value) | 0.401 | | 0.352 | | 0.426 | | 0.365 | |

This table reports estimates of Eq. (2) for the period 2010–2017 using a panel of rural areas in 32 Indonesian provinces. The dependent variable is the concentration index (CI) of under-5 mortality, where more negative values indicate a greater concentration of mortality among poorer households. All specifications are estimated using two-step system GMM with the first-difference transformation and include year fixed effects. Lagged values of the endogenous variables are used as internal instruments, while columns (1)–(4) differ in the inclusion of external instruments (log of own-source revenue and oil exports), as indicated by the checkmarks. Public health expenditure per capita is included in logarithms. Standard errors are Windmeijer-corrected for finite samples and reported in parentheses. The Arellano–Bond tests report p-values for first- and second-order serial correlation in first differences. The Hansen J-test reports the p-value for the null hypothesis that the instrument set is valid. The total number of instruments is reported for each specification.

*** p < 0.01, ** p < 0.05, * p < 0.1

**Table A6: Effect of public health expenditure on under-5 mortality rates**

|  | (1) | | (2) | | (3) | | (4) | |
| --- | --- | --- | --- | --- | --- | --- | --- | --- |
| VARIABLES | U5MR | | U5MR | | U5MR | | U5MR | |
| Total public health expenditure per capita | -0.011 | | -0.004 | | -0.012 | | -0.013 | |
|  | (0.008) | | (0.018) | | (0.012) | | (0.015) | |
| Household expenditure per capita | 0.033 | | 0.001 | | -0.013 | | 0.036 | |
|  | (0.034) | | (0.071) | | (0.245) | | (0.062) | |
| Poverty rate | 0.208* | | 0.105 | | 0.062 | | 0.205 | |
|  | (0.117) | | (0.201) | | (0.843) | | (0.197) | |
| Literacy rate | -0.003 | | 0.059 | | 0.085 | | -0.042 | |
|  | (0.147) | | (0.164) | | (0.600) | | (0.205) | |
| Electricity rate | 0.048 | | 0.055 | | 0.049 | | 0.037 | |
|  | (0.034) | | (0.052) | | (0.074) | | (0.059) | |
| Sanitation rate | -0.056* | | -0.055* | | -0.047 | | -0.058 | |
|  | (0.030) | | (0.031) | | (0.055) | | (0.034) | |
| Children immunisation | -0.104 | | -0.102 | | -0.086 | | -0.150 | |
|  | (0.132) | | (0.115) | | (0.408) | | (0.151) | |
| Lagged U5MR | -0.051 | | -0.056 | | -0.085 | | -0.072 | |
|  | (0.063) | | (0.066) | | (0.096) | | (0.056) | |
| Observations | 242 | | 242 | | 242 | | 242 | |
| Instruments  Log of own-source revenue | ✓ | | ✓ | |  | |  | |
| Oil exports | ✓ | |  | | ✓ | |  | |
| Lagged values of the endogenous variables | ✓ | | ✓ | | ✓ | | ✓ | |
| Total number of instruments | 30 | | 29 | | 28 | | 28 | |
| Number of provinces | 32 | | 32 | | 32 | | 32 | |
| Arellano–Bond AR (1) test in differences (p value) | 0.001 | 0.002 | | 0.006 | | 0.001 | |  |
| Arellano–Bond AR (2) test in differences (p value) | 0.262 | | 0.195 | | 0.222 | | 0.252 | |
| Hansen J-test (p value) | 0.742 | | 0.823 | | 0.721 | | 0.812 | |

This table reports estimates of Eq. (2) for the period 2010–2017 using a panel of 32 Indonesian provinces. The dependent variable is the under-5 mortality rate. All specifications are estimated using two-step system GMM with the first-difference transformation and include year fixed effects. Lagged values of the endogenous variables are used as internal instruments, while columns (1)–(4) differ in the inclusion of external instruments (log of own-source revenue and oil exports), as indicated by the checkmarks. Public health expenditure per capita is included in logarithms. Standard errors are Windmeijer-corrected for finite samples and reported in parentheses. The Arellano–Bond tests report p-values for first- and second-order serial correlation in first differences. The Hansen J-test reports the p-value for the null hypothesis that the instrument set is valid. The total number of instruments is reported for each specification.

*** p < 0.01, ** p < 0.05, * p < 0.1
